# Clinical Review of Ocular Traumas Resulting in Enucleation or Evisceration in a Tertiary Eye Care Center in Hungary

**DOI:** 10.1155/2021/5588977

**Published:** 2021-05-27

**Authors:** Gábor Tóth, Milán Tamás Pluzsik, Béla Csákány, Gábor László Sándor, Olga Lukáts, Zoltán Zsolt Nagy, Nóra Szentmáry

**Affiliations:** ^1^Dr. Rolf M. Schwiete Center for Limbal Stem Cell and Congenital Aniridia Research, Saarland University, Kirrberger Str. 100, D-66424 Homburg/Saar, Germany; ^2^Department of Ophthalmology, Semmelweis University, Budapest, Hungary; ^3^Department of Ophthalmology, Bajcsy-Zsilinszky Hospital, Budapest, Hungary

## Abstract

**Purpose:**

To analyse the demographic and clinical characteristics of ocular traumas resulting in enucleation/evisceration in a large tertiary referral center in a developed country (Hungary) over a period of 15 years. *Patients and Methods*. A retrospective review of enucleated/eviscerated eyes that underwent surgery between 2006 and 2020 at the Department of Ophthalmology of Semmelweis University, Budapest, Hungary, due to ocular trauma as the primary indication for enucleation/evisceration. For each subject, clinical history, B-scan ultrasound report, and histopathology results were reviewed.

**Results:**

There were 124 enucleated/eviscerated eyes from 124 patients (91 males (73.4%)). The mean age at the time of trauma was 37.3 ± 26.0 years while the mean age at the time of enucleation/evisceration was 46.9 ± 20.3 years. The main clinical diagnoses after ocular trauma were open globe injury (*n* = 96; 77.4%), ocular burns (*n* = 6; 4.8%), traumatic optic neuropathy (*n* = 4; 3.2%), bulbar avulsion (*n* = 3; 2.4%), traumatic cataract (*n* = 2; 1.6%), retinal ablation (*n* = 1; 0.8%), and traumatic carotid-cavernous fistula (*n* = 1; 0.8%). Among the 124 patients, 98 (79.0%) underwent enucleation and 26 (21.0%) evisceration. Patients who underwent primary enucleation/evisceration (*n* = 24 19.4%) were significantly older at the time of the injury (57.7 ± 22.7 years) than people who underwent secondary eye removal (32.4 ± 24.4 years) (*p* < 0.0001). The mean time interval between trauma and enucleation/evisceration was 114.9 ± 163.5 months. The main clinical indications for anophthalmic surgery were atrophia/phthisis bulbi (*n* = 56, 45.2%), acute trauma (*n* = 25, 20.2%), painful blind eye due to glaucoma (*n* = 17, 13.7%), endophthalmitis (*n* = 10, 8.1%), and cosmetic reasons (*n* = 7, 5.6%). One patient (0.8%) had sympathetic ophthalmia.

**Conclusions:**

Primary enucleation/evisceration was performed in one-fifth of all ocular trauma-related anophthalmic surgeries in our tertiary eye care center with enucleation being the most common procedure. Atrophia/phthisis bulbi was the most frequent immediate clinical indication for enucleation/evisceration.

## 1. Introduction

Ocular trauma is a leading indication for bulbar enucleation and evisceration worldwide [1]. Enucleation and evisceration are sometimes unavoidable end-stage solutions for several eye diseases. These may be required due to malignant tumours, after severe traumas, or in cases of painful blind eye [2].

Almost every third severe ocular injury leads to blindness in the USA [3]. Primary enucleation and evisceration should be avoided if there is a minimal chance to salvage the globe. Nevertheless, enucleation or evisceration (enucleation/evisceration) should be considered in cases with a visual acuity of no light perception (NLP) in order to prevent sympathetic ophthalmia or to reduce pain [4]. However, there are no available definitive guidelines when primary enucleation/evisceration should be performed in cases of ocular trauma [5].

Several previous studies have analysed ocular injuries that have resulted in enucleation/evisceration in different countries [5–7], but there is no current information available for European countries.

The primary aim of this study was to analyse the demographic and clinical characteristics of ocular traumas that resulted in enucleation/evisceration, as well as the frequency of enucleation versus evisceration, and the frequency of primary versus secondary enucleation/evisceration in a tertiary eye care center in a European developed country (Hungary) over a period of 15 years.

## 2. Materials and Methods

This retrospective study was undertaken at a tertiary eye care center to analyse demographic and clinical characteristics of patients who suffered ocular trauma and later underwent enucleation/evisceration in Hungary. The study was approved by the Regional and Institutional Committee of Science and Research Ethics of Semmelweis University, Hungary (No. 243/2020). The study was performed in accordance with the Declaration of Helsinki Guidelines for Human Research.

In total, 729 eyes underwent enucleation/evisceration over a period of 15 years (between January 2006 and December 2020) at the Department of Ophthalmology of Semmelweis University. Among these, 124 eyes of 124 patients were removed due to ocular trauma. Our study was conducted on these 124 eyes.

For each subject, the clinical data were reviewed including patient demographics, clinical history, presenting visual acuity at the time of ocular injury and enucleation/evisceration, clinical indication for enucleation/evisceration, B-scan ultrasound reports (Ultrascan with 10 MHz and 20 MHz ultrasound probes, Alcon, USA), operative details, and results of the histopathological analysis following enucleation/evisceration. Paraffin sections stained with haematoxylin-eosin and histopathological charts were available for 119 (96.0%) globes. Visual acuity data were converted from Snellen chart values to LogMAR format.

Our study was performed in a retrospective fashion and many patients were referred to our clinic only weeks, months, or years after the trauma, as our department is a tertiary eye care center. Therefore, we could not examine the complete course of all cases, as documentations were incomplete. As a result, this classification could not be performed either using the Birmingham eye trauma terminology system [8] or the Shukla's ocular trauma classification system [9] for the present study.

Statistical analysis was performed with Statistica 8.0 (StatSoft Inc., Tulsa, OK, USA). Data were expressed as mean with standard deviation (SD). Chi square test was used for differences in proportions of categorical variables. To compare the two groups regarding age, time interval between trauma and enucleation/evisceration, visual acuity, number of preexisting ocular factors and systemic diseases, and number of surgeries, we used the student's t-test in case of normal distribution and the nonparametric Mann–Whitney U test for not normally distributed variables. A *p* value lower than 0.05 was considered statistically significant.

## 3. Results

**Figure 1:**
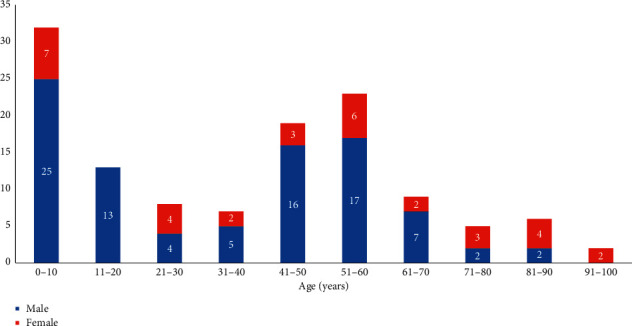
Age and sex distribution of patients at the time of the ocular injury.

**Figure 2:**
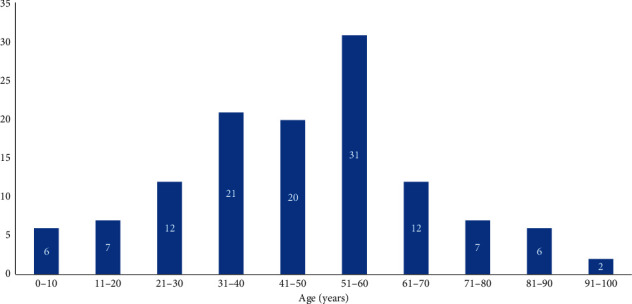
Age distribution of patients at the time of the enucleation/evisceration.

The demographic and medical characteristics and age distribution of the patients are shown in Table 1 and Figures 1 and 2. There was no significant difference (*p*=0.148) regarding proportion of primary enucleations and eviscerations. There was no significant difference between primary and secondary enucleation/evisceration groups regarding sex (*p*=0.938) and laterality (*p*=0.415). The mean age of the patients was 37.3 ± 26.0 years (range, 1 to 100 years) at the time of ocular trauma and 46.9 ± 20.3 years (range, 3 to 100 years) at the time of enucleation/evisceration. Twenty-four subjects (19.4%) had primary and 100 (80.6%) had secondary enucleation/evisceration. Patients who underwent primary enucleation/evisceration (*n* = 24 19.4%) were significantly older at the time of the injury (57.7 ± 22.7 years) than people who underwent secondary eye removal (32.4 ± 24.4 years) (*p* < 0.0001). Among patients with primary enucleation/evisceration, best corrected distance visual acuity was NLP in 20 (83.3%) cases and light perception (LP) in 4 (16.7%) cases.

Associated preexisting systemic diseases, social factors, and preexisting ophthalmological factors are shown in Table 2.

The main clinical diagnoses after ocular trauma were (in decreasing order) open globe injury (*n* = 96; 77.4%), ocular burns (*n* = 6; 4.8%), traumatic optic neuropathy (*n* = 4; 3.2%), bulbar avulsion (*n* = 3; 2.4%), traumatic cataract (*n* = 2; 1.6%), retinal ablation (*n* = 1; 0.8%), traumatic carotid-cavernous fistula (*n* = 1; 0.8%), and unclassifiable due to incomplete documentation (*n* = 11; 8.9%). In the open globe injury group, 47 eyes (49.0%) had a corneal, 26 (27.1%) had a scleral, and 23 (23.9%) had a corneoscleral laceration.

The injury occurred at home in 57 cases (46.0%), at the workplace in 17 cases (13.7%), in a violent act in 13 cases (10.5%), in a traffic accident in seven cases (5.6%), and in a sports accident in three cases (2.4%). The circumstances of the injury were not determinable in 27 (21.8%) cases. Gunshot injury occurred in two cases (1.6%), both of them at home, as an accident. Intraocular foreign body was found in 17 (13.7%) and intraorbital foreign body was detectable in 2 (1.6%) eyes. Primary enucleation/evisceration was performed within 24 hours in 21 patients (16.9%) and within one week in three cases (2.4%).

Most frequently performed ocular surgeries in order to manage ocular trauma before enucleation/evisceration are shown in Table 3.

Immediate clinical indications for enucleation/evisceration (last diagnosis before anophthalmic surgery) are displayed in Table 4.

Among four patients who underwent primary enucleation/evisceration with a visual acuity of LP, two had total corneal melting with endophthalmitis, one had total corneal melting without endophthalmitis, and one had buphthalmos with extensive scleral laceration.

B-scan ultrasound examinations showed retinal detachment in 34 (27.4%) eyes, atrophia/phthisis bulbi in 29 (23.4%), choroidal detachment in 13 (10.5%), normal posterior segment in 12 (9.7%), vitreous haemorrhage in 11 (8.9%), silicon oil in 9 (7.3%), endophthalmitis in 7 (5.6%), optic disc cupping in 4 (3.2%), subretinal haemorrhage in 2 (1.6%), mild vitreous inflammation in 2 (1.6%), suprachoroideal haemorrhage in 1 (0.8%), optic nerve damage in 1 (0.8%), crystalline lens dislocation in 1 (0.8%), empty scleral shell in 1 (0.8%), and intravitreal foreign body in 1 (0.8%). B-scan reports were not available in 25 (20.2%) cases.

Histopathological diagnoses after enucleation/evisceration are shown in Table 5.

Only one patient (0.8%) had sympathetic ophthalmia in our sample, who was referred to our clinic with granulomatous panuveitis on the right eye and phthisis bulbi on the left eye 50 years following open globe injury on the left eye.

Among the 124 patients who underwent enucleation/evisceration, 50 (40.3%) received a primary orbital implant at the time of eye removal. The type of implant was silicone in 26 (52.0%), aluminium-oxide ceramic in 5 (10.0%), and hydroxyapatite in 19 (38.0%) cases.

## 4. Discussion

We analysed the demographic, etiological, and clinical details of ocular trauma cases that led to loss of the eye over a 15-year period at a tertiary eye care center in Hungary. To the best of our knowledge, this is the first study to report on the characteristics of ocular injuries leading to enucleation/evisceration in Europe.

The incidence of ocular trauma leading to enucleation is reported to be 1.1% among patients with ocular trauma in China [10]. Ocular trauma is the second most frequent primary indication for enucleation following tumours in Hungary [11].

In our sample, mean age at the time of enucleation/evisceration due to ocular trauma (46.9 years) was similar to the findings of de Gottrau et al. in Germany [12] (44.2 years) and Gauthier et al. [5] in the USA (45.2 years). Predominance of males (73.4%) was also similar to the international trends: 79% in Germany [12], 80.9% in Turkey [13], 81.3% in China, [14] and 75% in the USA [6]. Male predominance at younger age may be associated with occupational exposure and risk-taking behavior (higher incidence of assault, sport, and traffic accident-related ocular injuries) [15]. However, a change in male/female proportion was observable in older ages, as we found a female predominance (69.2%) in people older than 70 years, similar to the study by Vidne-Hay et al. [16], which may be related to the suggestion that elderly women with osteoporosis have an increased risk of falling and sustaining injuries [17].

Similar to the findings of Gauthier et al. in the USA (27.2%) [5], primary enucleations/eviscerations were performed less frequently (19.4%) in our department than secondary anophthalmic surgeries (80.6%). Mean age of the patients with primary enucleation/evisceration at the time of trauma in our study (57.7 years) is similar to the finding by Gauthier et al. in the USA (53.8 years) [5]. Regarding our sample, patients with primary enucleation/evisceration were older (57.7 years) than patients with secondary enucleation/evisceration (32.4 years). Increased age is a known associated factor for primary enucleation/evisceration, as general anesthesia during multiple procedures may mean a risk for elderly people [18]. Secondary enucleation/evisceration was preferred in every case in our department where there was a minimal chance for visual recovery or to salvage the globe.

Similar to the study by Sigurdsson et al. in Iceland [19], most of the injuries occurred at home and at the workplace with assault being uncommon in our sample. In contrast, assault and gunshot accidents represent the most common etiological factors in trauma-related anophthalmic surgeries in the USA, as reported by Gauthier et al. [5]. This difference can be explained with the different firearm regulations in the USA and Hungary, as purchase of handguns is very strictly regulated in Hungary. There were only two cases with air gun injury in our study population. Open globe injuries were found to be the most prevalent ocular traumas resulting in enucleation/evisceration in both countries.

The order of frequency of most common immediate clinical indications and pathological diagnoses for enucleation/evisceration in our sample were the same as reported by de Gottrau et al. (atrophia bulbi, acute trauma, secondary glaucoma, and endophthalmitis) in Germany [12] thirty years ago. Enucleation was also mainly performed as a secondary procedure in their study, similar to our present report.

Preference for enucleation over evisceration may be related to the well-known supposition in the international literature that evisceration does not eliminate the risk of sympathetic ophthalmia after ocular trauma [20]. Nevertheless, we did not have any case of sympathetic ophthalmia in patients who underwent evisceration. Only one case of sympathetic ophthalmia was diagnosed in a patient who underwent enucleation 50 years following an open globe injury. In this case, the diagnosis could not be histologically proven. Similarly, in previous reports, sympathetic ophthalmia could histologically only be confirmed in half of the cases and remained a clinical diagnosis [21]. The incidence of sympathetic ophthalmia in our report (0.8%) is slightly higher than 0% reported by Gauthier et al. in the USA [5].

Similar to evisceration, enucleation was also most commonly performed as a secondary procedure in our patients. Ophthalmologists are rather careful and tend to avoid enucleation as a primary procedure after ocular trauma, as every eye deserves a chance.

In the present study, the number of known preexisting systemic and ocular diseases was ten times lower in patients with trauma than in subjects with enucleation/evisceration due to corneal ulcer in the same institution, in a previous study [22]. This may be related to the lower age of subjects with trauma (37.3 years) compared to those with corneal ulcer (66.4 years) in the previous study. Patients with psychiatric disorders are exceptions, as they seem to have more systemic diseases even in younger age, at least in our study population with ocular trauma.

Limitations of our study include that it was performed in a retrospective fashion; our department is a tertiary eye care center, and for this reason, we could not examine the complete course of these cases and many patients were only referred to our clinic when the eyes could no longer be salvaged.

## 5. Conclusions

Secondary enucleation/evisceration was performed more frequently after ocular trauma in our department than primary anophthalmic surgery. Atrophia/phthisis bulbi was the most frequent immediate clinical indication for enucleation/evisceration.

## Figures and Tables

**Table 1:** Demographic and medical characteristics of patients with enucleation or evisceration due to ocular trauma.

	Total eyes (*n* = 124)	Primary enucleation/evisceration	Secondary enucleation/evisceration	*P* value
Procedure, *n* (%)				0.148
(i) Enucleation	98 (79.0)	22 (91.7)	76 (76.0)	
(ii) Evisceration	26 (21.0)	2 (8.3)	24 (24.0)	

Sex, *n* (%)				0.938
(i) Male	91 (73.4)	18 (75.0)	73 (73.0)	
(ii) Female	33 (26.6)	6 (25.0)	27 (27.0)	

Laterality, *n* (%)				0.415
(i) Right	62 (50.0)	9 (37.5)	53 (53.0)	
(ii) Left	62 (50.0)	15 (62.5)	47 (47.0)	

Age at the time of the trauma, years (mean ± SD (range))	37.3 ± 26.0 (1–100)	57.7 ± 22.7 (10–100)	32.4 ± 24.4 (1–87)	**<0.00001**

Age at the time of enucleation/evisceration, years (mean ± SD)	46.9 ± 20.3 (3–100)	57.7 ± 22.7 (10–100)	44.2 ± 18.9 (3–87)	**0.0073**

Time between trauma and enucleation/evisceration, months (mean ± SD (range))	114.9 ± 163.5 (0.3–840)	0.04 ± 0.04 (0.03–0.23)	142.5 ± 171.0 (0.1–840)	**<0.00001**

Best corrected distance visual acuity (LogMAR) at the time of trauma (mean ± SD (range))	2.69 ± 0.65 (0.10–3.00)	2.97 ± 0.09 (2.70–3.00)	2.52 ± 0.77 (0.10–3.00)	0.052

Best corrected distance visual acuity (LogMAR) at the time of enucleation/evisceration (mean ± SD (range))	2.94 ± 0.14 (2.30–3.00)	2.95 ± 0.11 (2.70–3.00)	2.93 ± 0.15 (2.30–3.00)	0.865

Number of preexisting ocular factors at the time of the trauma, *n* (mean ± SD (range))	1.1 ± 0.4 (0–2)	1.2 ± 0.7 (0–2)	1.1 ± 0.3 (0–2)	0.362

Number of preexisting systemic diseases and social factors at the time of the trauma, *n* (mean ± SD (range))	1.2 ± 0.8 (0–5)	1.3 ± 0.8 (0–2)	1.3 ± 0.8 (0–5)	0.161

Average number of surgeries between trauma and enucleation/evisceration, *n* (mean ± SD (range))	1.6 ± 1.6 (0–8)	0	2.0 ± 1.6 (0–8)	**<0.00001**

**Table 2:** Associated preexisting ophthalmological factors, systemic diseases, and social factors at the time of ocular trauma for patients who underwent secondary enucleation or evisceration.

Ophthalmological factors	*N*	Systemic diseases and social factors	*N*
Previous cataract surgery	6	Hypertension	17
Glaucoma (with buphthalmos)	5 (3)	Diabetes mellitus	11
Endocrine orbitopathy	1	Psychiatric disorders	5
Previous ocular trauma	1	Etilism	5
Previous corneal transplantation	1	Dementia	4
Amblyopia	1	Cardiac disease	4
Previous trachoma	1	Hypo/hyperthyroidism	3
		Homelessness	2
		Chronic vertigo	1

**Table 3:** Ocular surgeries between ocular trauma and secondary enucleation/evisceration.

Ocular surgeries	*N*
Corneal wound closure	48
Scleral wound closure	36
Cataract surgery	34
Vitrectomy	32
Keratoplasty	13
Glaucoma surgery	12
Amniotic membrane transplantation	5
Conjunctival autograft transplantation	4
Tarsorrhaphy	2
Corneal abrasion	2
Eye muscle surgery	2
Canthotomy	2
Iris reconstruction	1
Anterior chamber paracentesis	1
Phototherapeutic keratectomy	1
Limbal stem cell transplantation	1
Keratoprosthesis implantation	1

**Table 4:** Immediate clinical indications for enucleation/evisceration due to ocular trauma (last diagnosis before anophthalmic surgery).

Immediate clinical indications	*N* (%)
Atrophia/phthisis bulbi	56 (45.2)
Acute trauma	25 (20.2)
Painful blind eye due to glaucoma	17 (13.7)
Endophthalmitis	10 (8.1)
Cosmetic reasons	7 (5.6)
Corneal ulcer	4 (3.2)
Threatening perforation	2 (1.6)
Wound dehiscence after ocular reinjury	2 (1.6)
Keratoprosthesis extrusion	1 (0.8)
Total	124 (100)

**Table 5:** Histopathological diagnoses for enucleated/eviscerated eyes due to ocular trauma.

Histopathological diagnoses	*N* (%)
Atrophia/phthisis bulbi	55 (44.4)
Corneal/scleral wound	22 (17.7)
Secondary glaucoma	19 (15.3)
Corneal ulcer	9 (7.3)
Endophthalmitis	8 (6.5)
Conjunctival epithelial ingrowth	3 (2.4)
Earlier keratoplasty	1 (0.8)
Chronic uveitis	1 (0.8)
Descemetocele	1 (0.8)
Unavailable	5 (4.0)
Total	124 (100)

## Data Availability

All data analysed during this study are included in this article.
